# Supplementary material for: Sedimentary biomarkers of human presence and taro cultivation reveal early horticulture in Remote Oceania
Source: Commun Earth Environ. 2024 Nov 6;5(1):667. doi: 10.1038/s43247-024-01831-8 (PMC11541199; doi:10.1038/s43247-024-01831-8)
Supplement: Supplementary file 2 — Supplementary material [file 43247_2024_1831_MOESM2_ESM.pdf]

Supplementary information for

Sedimentary biomarkers of human presence and taro cultivation reveal early horticulture in Remote Oceania

Authors

Giorgia Camperio[1,2]*, S. Nemiah Ladd[3], Matiu Prebble[4,5], Ronald Lloren[1,2], Elena Argiriadis[6,7], Daniel B. Nelson[8], Christiane Krentscher[1], Nathalie Dubois[1,2]

Affiliations

[1]Department of Surface Waters Research & Management, Eawag, Dübendorf, Switzerland.

[2]Department of Earth Sciences, ETH Zürich, Zürich, Switzerland

[3]Department of Environmental Sciences, University of Basel, Basel, Switzerland

[4]School.

of Earth and Environment, College of Science, University of Canterbury, Christchurch, New Zealand.

[5]Archaeology and Natural History, Culture History and Languages, The Australian National University, Canberra, Australia.

[6]Institute of Polar Sciences, CNR-ISP, Venice, Italy.

[7]Department of Environmental Sciences, Informatics and Statistics, Ca' Foscari University, Venice, Italy.

[8]Department of Environmental Sciences – Botany, University of Basel, Basel, Switzerland.

*Corresponding author, giorgia.camperio@eawag.ch

This PDF file includes:

Supplementary Text S1 to S3
Figs. S1 to S10
Tables S1 to S2
References

Supplementary Text

Supplementary Text 1

Assessing the robustness of the precipitation signal in leaf wax δ^2H values in the Emaotfer record

We interpret the δ^2H values of longer chain *n*-alkanoic acids (*n*-C26, *n*-C28, *n*-C30) in the Emaotfer sedimentary record as being indicative of local precipitation δ^2H values[18,19], which are strongly inversely correlated with precipitation amount at low-elevation coastal sites in the tropical Pacific[20-22]. While other factors can affect the δ^2H values of both precipitation and leaf waxes[23-27], this interpretation seems most probable for the Emaotfer record, as explored below.

First, as a low-elevation site away from major landmasses in the tropical Pacific, Emaotfer is a typical location where the so-called "amount effect" (*i.e.*, lower precipitation δ^2H values during rainier periods) has been empirically observed through data from the Global Network of Isotopes in Precipitation (GNIP) and in isotope-enabled atmospheric models[22]. The amount effect can be explained by two main mechanisms: the re-evaporation of falling rain and diffusive exchanges with surrounding vapour in low precipitation regimes, and the recycling of subcloud layer vapour feeding convective systems in high precipitation regimes[28]. While orographic effects play a key role in determining precipitation isotopes on continents or at high elevation sites including islands in the Pacific[29], these are less relevant at our windward, low-elevation coastal site on the island of Efate.

Second, it is necessary to consider how well variations in precipitation δ^2H values are recorded by the δ^2H values of long-chain *n*-alkanoic acids in our sedimentary record, as changes in the vegetation source can lead to different isotope fractionations[23-25]. Although δ^2H values of longer chain *n*-alkanoic acids (*n*-C26, *n*-C28, *n*-C30) covaried (Fig. S6), for the interpretation of hydroclimatic variations, we use the δ^2H values of *n*-C30, as this compound is most unambiguously associated with higher plants[30-32]. Lipid concentrations of modern plants collected in Vanuatu (Fig. S7) confirm that only few species produce *n*-C30 in the archipelago: The main producers are *Calophyllum inophyllum*, an indigenous tree that has the highest concentrations of *n*-C30, followed by the trees *Tectona grandis*, and *Burkella obovata*. As *n*-C30 is only sourced from a relatively small number of terrestrial trees in this context, its δ^2H values are less susceptible to changes in plant community composition, such as the ones expected after land clearing for agriculture.

If the δ^2H values of *n*-alkanoic acids are influenced by changes in vegetation source, this should also be apparent in the relative hydrogen isotope offsets among *n*-alkanoic acids, which can be expressed as ε values ($\varepsilon = [(\delta Lipid\_1 +1)/(\delta Lipid\_2 +1)] - 1)$[33], as different plants produce different amounts of each homologue (Fig S7). The ε values between *n*-C30 δ^2H values and those of other homologues, support the use of long chain-alkanoic acids in reconstructing hydroclimate in the setting, and also indicate how changes in evapotranspiration may have influenced the δ^2H values of some homologues (Figs S8, S9). During intervals when the swamp is interpreted to have had a continuous flow of water through it, and therefore comparatively less evaporative enrichment, the source of water for aquatic plants, represented by *n*-C22 and *n*-C24 (Fig. S9), would have been 2H-depleted relative to the leaf water in terrestrial plants. Epsilon C30/22 values are positive, which is expected if *n*-C30 was primarily derived from trees and *n*-C22 was from a mix of terrestrial and aquatic sources (here the aquatic component makes the net δ^2H value for *n*-C22 lower). During intervals when the swamp is interpreted to have been disconnected from the river, the fatty acid δ^2H values converge (ε values trend to 0 ‰), since the swamp water and leaf water would have both been 2H-enriched as a result of evaporation and transpiration, respectively, while both being fed by the same input water with a common precipitation isotope signal. Increases in precipitation amount during such drier periods would have also shifted the source water δ^2H values of both aquatic and terrestrial biomarkers higher, which would drive biomarker δ^2H values in the same direction as increased evapotranspiration during dry periods. The combined influences on expected δ^2H values therefore work together to indicate overall wetter/drier conditions, albeit without the capacity for direct quantitative inference. By comparison, the *n*-C28 δ^2H values show greater variance than those of *n*-C30, and this corresponds with wildly fluctuating ε values for *n*-C28/*n*-30, *n*-C28/*n*-24, *n*-C28/n-22 (Fig S9). This could be indicative of variable contributions from different plant sources that are not consistent over time, and makes the *n*-C28 less reliable than *n*-C30 for interpreting a primary hydroclimate signal. Nevertheless, *n*-C28 δ^2H values are positively correlated with *n*-C30 δ^2H values (Fig. S6), indicating a mainly terrestrial source, and overall supporting the use of n-C30 δ^2H values as a wetter/drier indicator. Furthermore, the comparison of ε for n-C28/n-C30 between the dry and wet phase is non significant (ns) further supporting their use as hydroclimatic indicators (Fig. S8). In conclusion, we interpret the δ^2H of longer chain *n*-alkanoic acids in the Emaotfer sedimentary record as reflecting primarily changes in the amount of precipitation.

Supplementary Text 2

XRF elemental data

In these settings, Al, Fe, and Ti reflect terrigenous input[1] from the surrounding catchment, (including pumice and alluvial deposits). Calcium input is normalised against aluminium (Ca/Al) to reflect the biogenic inputs of Ca from the swamp catchment located on an uplifted limestone mainly composed of CaCO3 rather than detrital Ca delivered from the surrounding catchment[1]. Interestingly, manganese records a second though smaller peak at this transition, reflecting a second change in the redox parameters in the system with an increase in oxygen that would be consistent with an hydrographic change to the system (Fig. S3). Wirrmann *et al.*[2] suggest that the nearby Teouma River likely contributed most of this terrigenous material. A recent LIDAR survey of Efate, however, reveals that the lowest elevation connection, a small 400m-wide saddle, sits at ~10 m above sea level (pers. comm. Phillip Parton). Flood waters could have potentially overtopped this saddle or directed terrigenous sediments back up through solutional conduits formed during mid-Holocene marine high stand conditions.

Supplementary Text 3

Sterols and palmitone

Gas Chromatography-Mass Spectrometry (GC-MS) measurements in Selected Ion Monitoring (SIM) mode were carried out for quantification. External standards (Table S2) were used to verify peak identity and quantification via external calibration curve. For analyses of sterol and stanol the initial column temperature program was 150°C (held 1 min), 1st ramp to 220°C at 40°C min-1, and 2nd ramp to 300°C at 3°C min-1 (held 5 min). For analyses of palmitone the initial column temperature program was 70°C (held 1. min) and a 1st ramp to 300°C at 20°C min-1 (held 15 min).

Faecal molecules have been previously applied in sedimentary contexts to trace human presence and demography[3–8]. While coprostanol and epicoprostanol are not unique to humans, they are always associated with their presence on Pacific Islands as they are produced by pigs and other omnivores[9] that were introduced to the Remote Pacific islands by humans[10], strengthening their potential as tracers of human presence in this setting.

Ratios of faecal sterols are often reported in literature to discriminate against producers of molecules [9], but hold limited potential in this tropical setting given the disproportionate amount of cholestanol deriving from the environmental degradation of cholesterol[11]. Most of the ratios involve the use of cholestanol to correct for independent cholesterol degradation in the environment. However, the application of these ratios in tropical peats have not been tested. We tested the most used ratio of copr + epi / (copr+epi+cholestanol) (Fig. S5). For this ratio, values above 3 are interpreted as faecal sources and values above 9 are associated with humans or pigs[8]. Despite the ratio increasing during the periods of human occupation starting at 181 cm the highest value of 6.8 is reached at 307 cm (3601-3810 BP). The low concentrations of cholestanol during the uplift phase could explain such peaks in the bottom of the core, which cannot be interpreted as human presence given the low quantities of coprostanol and epicoprostanol at this time. The values of cholestanol compared to its main cholesterol source could also hint to other unidentified sterol sources. With degradation, sterols progressively lose double bonds, so the ratio of a sterol to its corresponding stanol could be an indication of down-core degradation of biomarkers in the swamp[12]. However, unconstrained factors can influence the results, *e.g.* trophic conditions[13] and taxonomic differences of living organisms, including the occurrence of stanols in various living organisms[14].

A constant minimum concentration of faecal sterols is present throughout the whole core (mean 0.1153 ± 0.091 µg/g), indicating inputs from other possible producers of faecal sterols

that could be linked to microbial activity[15] or to the presence of other omnivores producing low quantities of faecal sterols (e.g. bats, birds, marine mammals)[16,17]. Constant quantities of palmitone are also present in the marine phase of the basin (mean 1.129 ± 0.4411 µg/g) indicating the possible production of this molecule by marine organisms. However, during the initial lake phase, palmitone is only present in low quantities (mean 0.584 ± 0.4380 µg/g) until 180 cm.

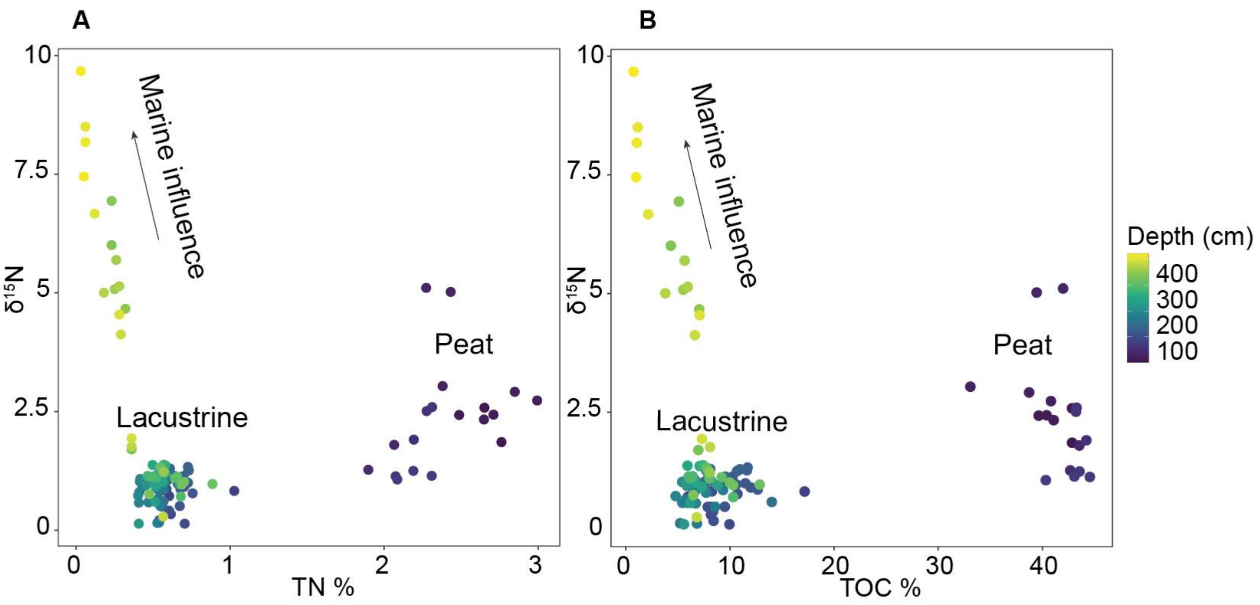

Fig. S1. Bulk sediment geochemistry of the Emaotfer sediment core showing its three main depositional phases. A) Crossplot of δ^{15}N of bulk sediment against TN%, B) crossplot of δ^{15}N and TOC%. Colour indicates the depth of samples.Higher δ15 in the upper left grouping reflects the possible marine influence at the bottom of the core with linked to the coastal uplift and post mid-Holocene sea level recession, the bottom left grouping represents the lacustrine phase, and the bottom right the peat part of the core.

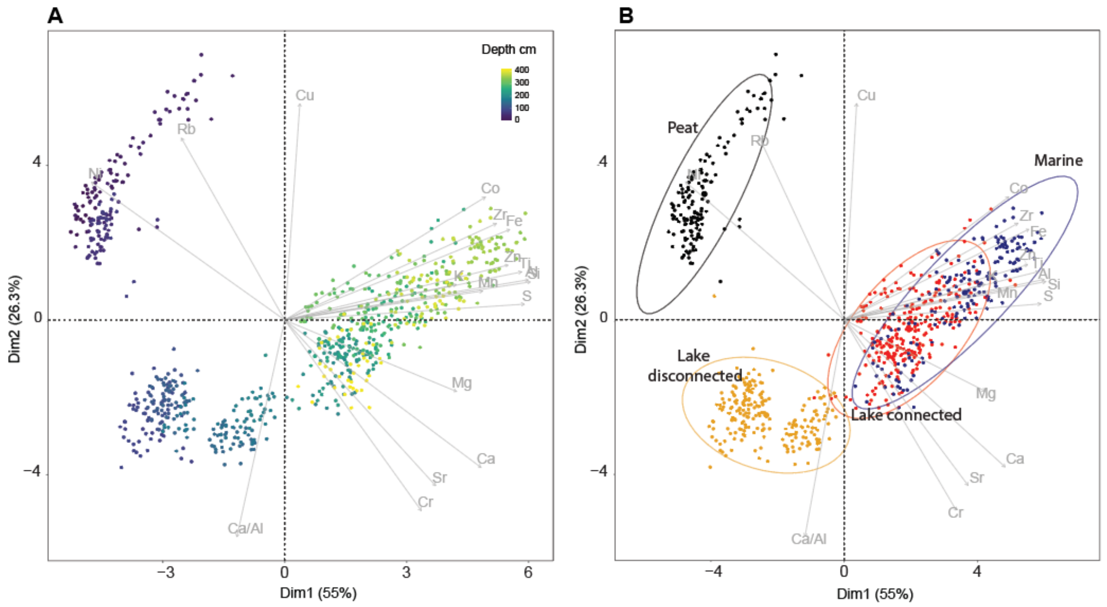

Fig. S2. Principal component analysis of the downcore XRF elemental distribution. Arrows indicate the contribution of each element. (A) Colour indicates the depth of samples. (B) Depositional phases are circled: peat (black), lake disconnected from the Teouma river (orange), lake connected (red), and marine (dark blue) phase (high terrestrial elements).

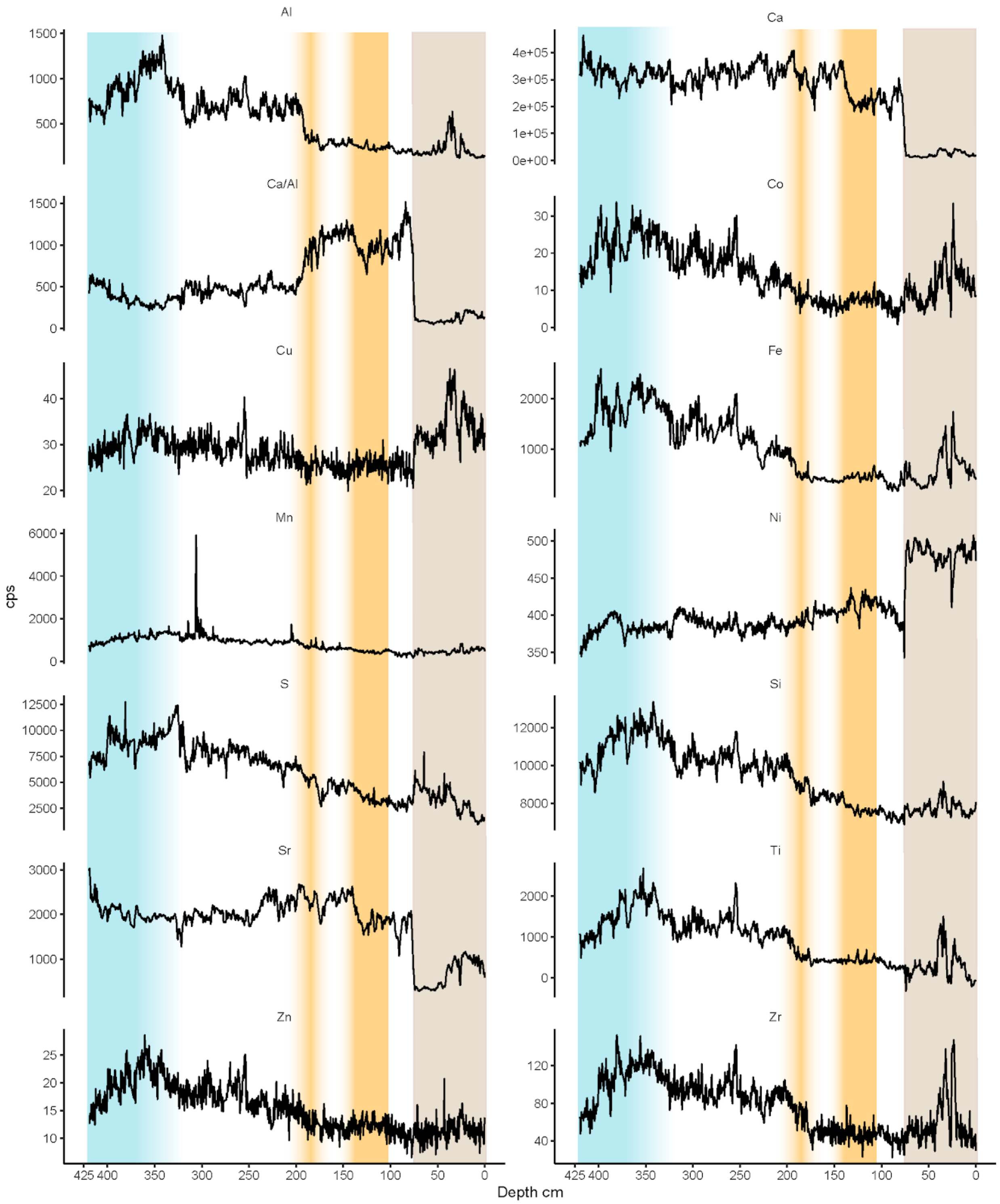

Fig. S3. Downcore XRF elemental distribution in counts per seconds (cps). Light blue shading represents the marine period of the basin, yellow shadings represent periods of human occupation corresponding to the Lapita and Erueti phase in the archaeological record, brown shading indicates the peat part of the core.

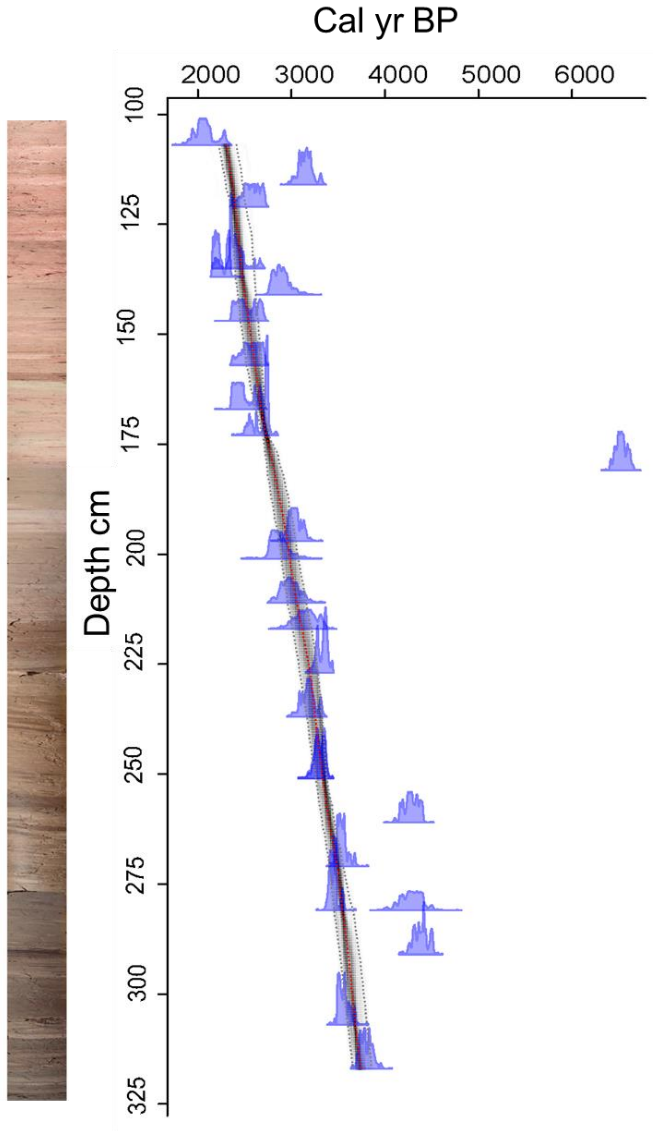

Fig. S4. Additional separate detailed age-depth model from 107 cm to 307 cm, refining the period of first human presence and excluding the peat and marine phase of the core. Age model obtained with rbacon r package 3.0.0[34]. Although this separate age model does not differ much from the full chronology (average difference of 15 years), it was used for the interpretation of the record in this section.

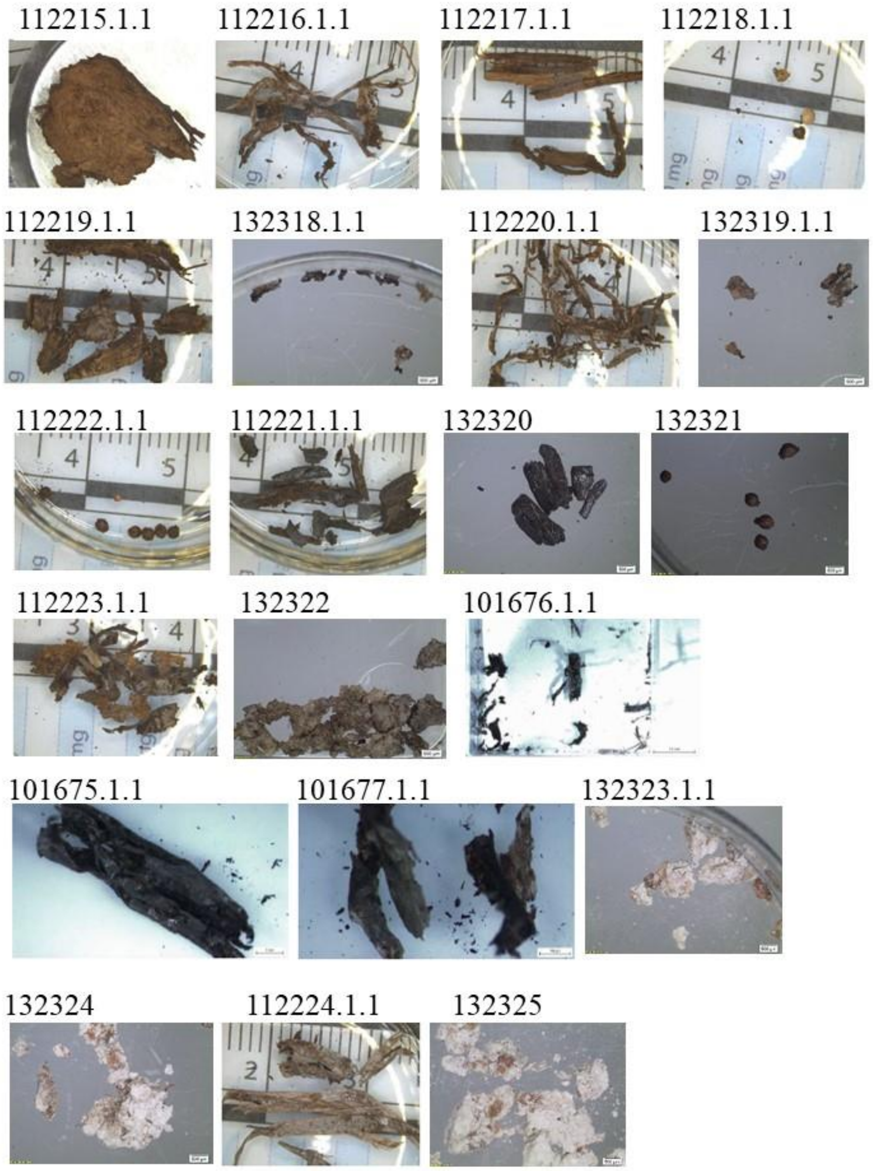

Fig, S5. Macrofossil images captured under the microscope, each identified by their respective lab codes. These remains were radiocarbon dated (Table S1). The majority of the dated materials consist of unidentifiable leaf and root fragments (plant remains), primarily

from monocotyledons, including *Cyperaceae* and *Pandanaceae*. These materials are considered to be short-lived with limited in-built age potential.

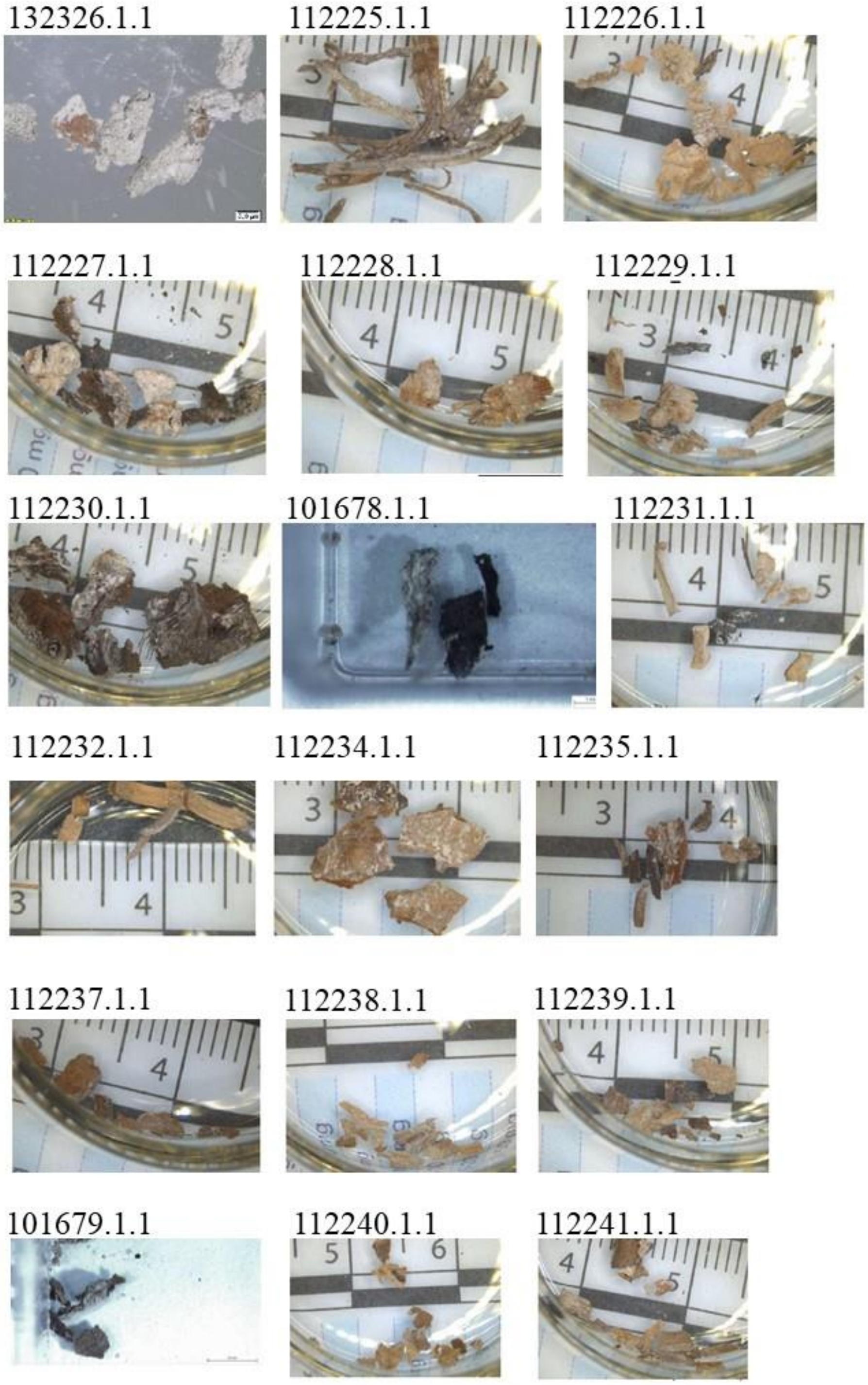

Fig. S5 continued.

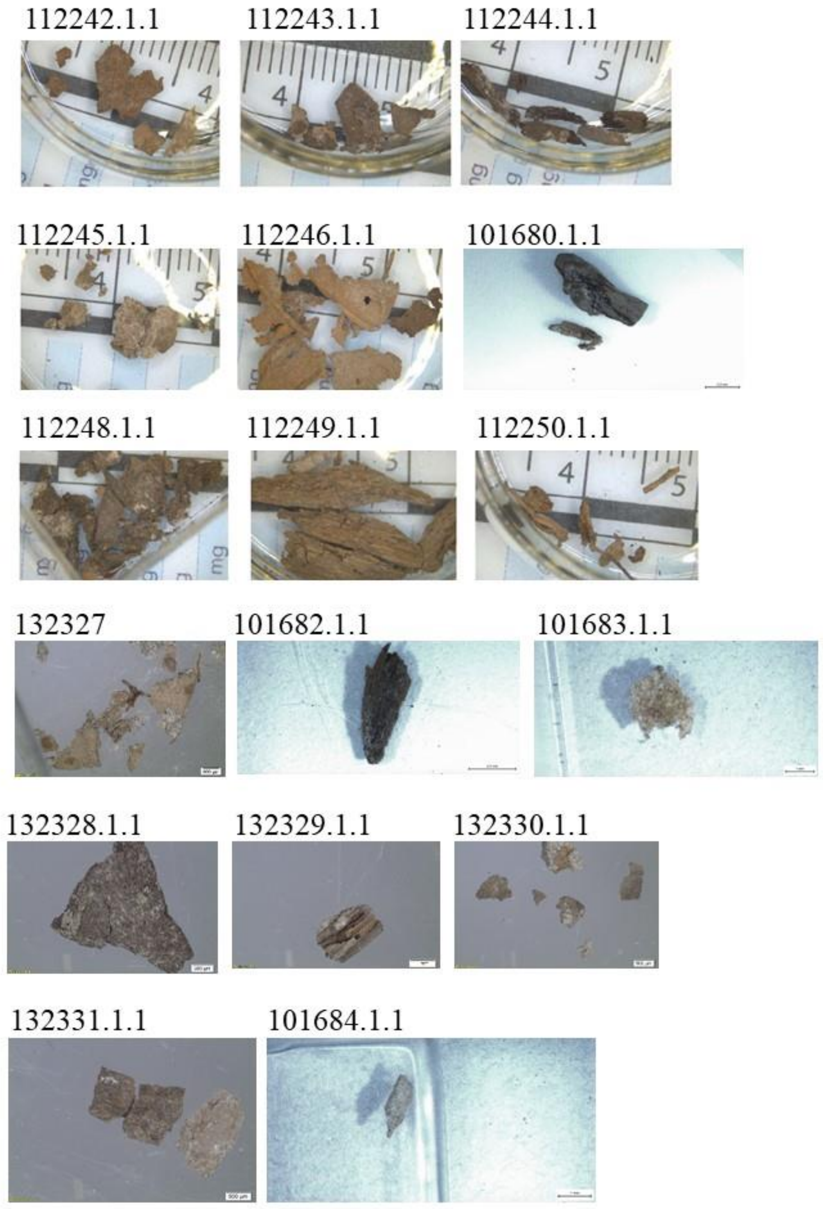

Fig. S5 continued.

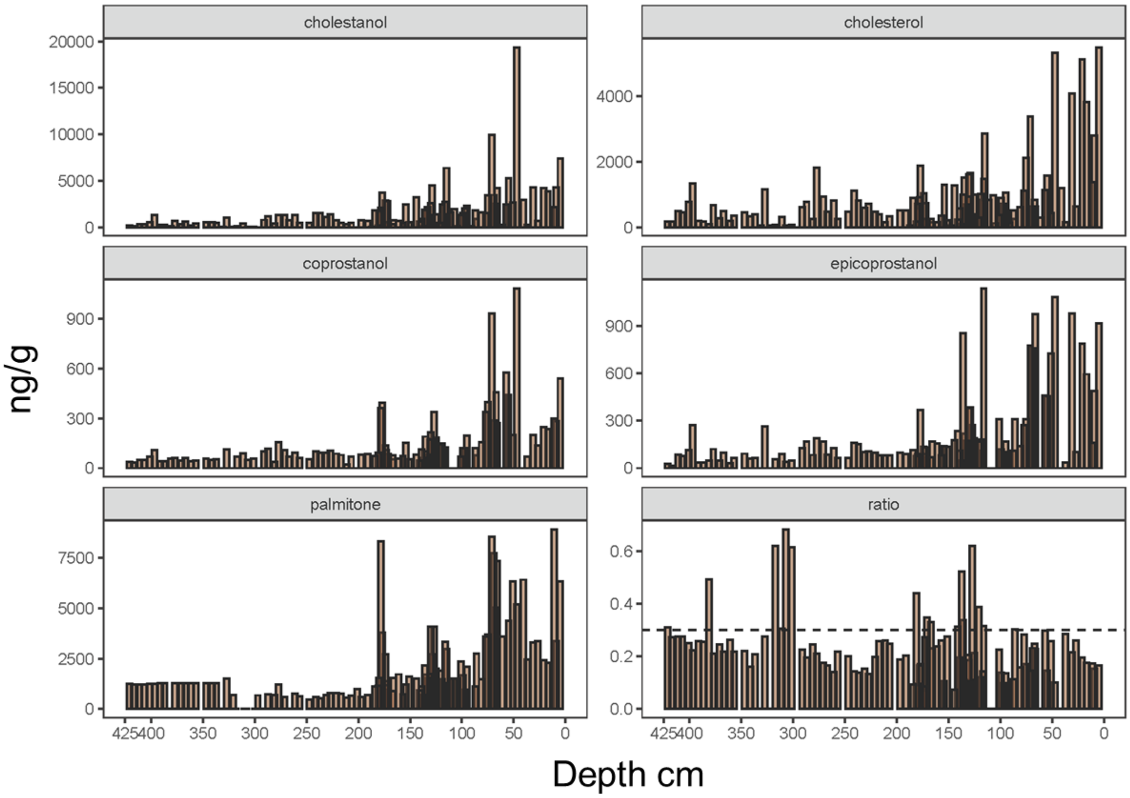

Fig. S6. Detailed downcore distribution of all the sterols and stanols used in this study, including the faecal ratio detailed in the text.

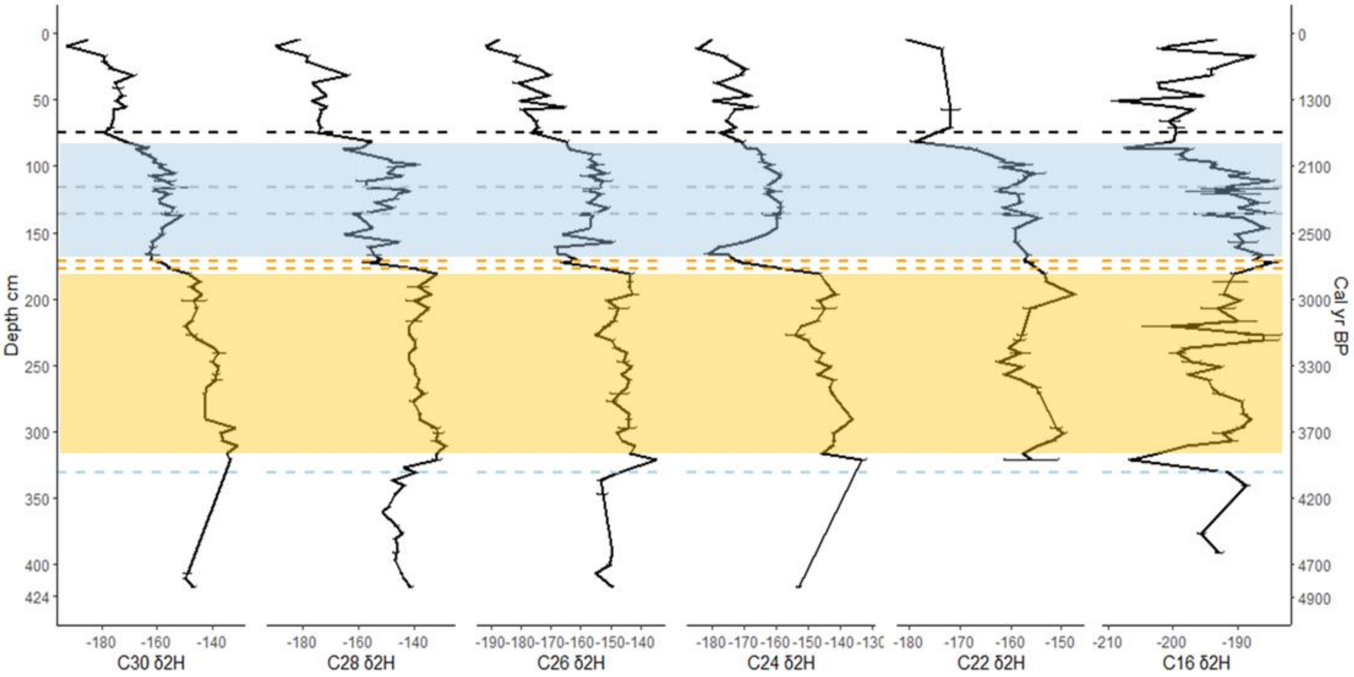

Fig. S7. Downcore hydrogen isotopic composition of longer chain *n*-alkanoic acids in the Emaotfer record. The blue shading illustrates the wetter interval characterising the time following initial settlement, in comparison to the preceding drier period illustrated by the yellow shading. Dashed black lines indicate the onset of the peat, grey dashed lines correspond to the Erueti period and orange dashed to the Lapita period as identified by palmitone and faecal sterols.

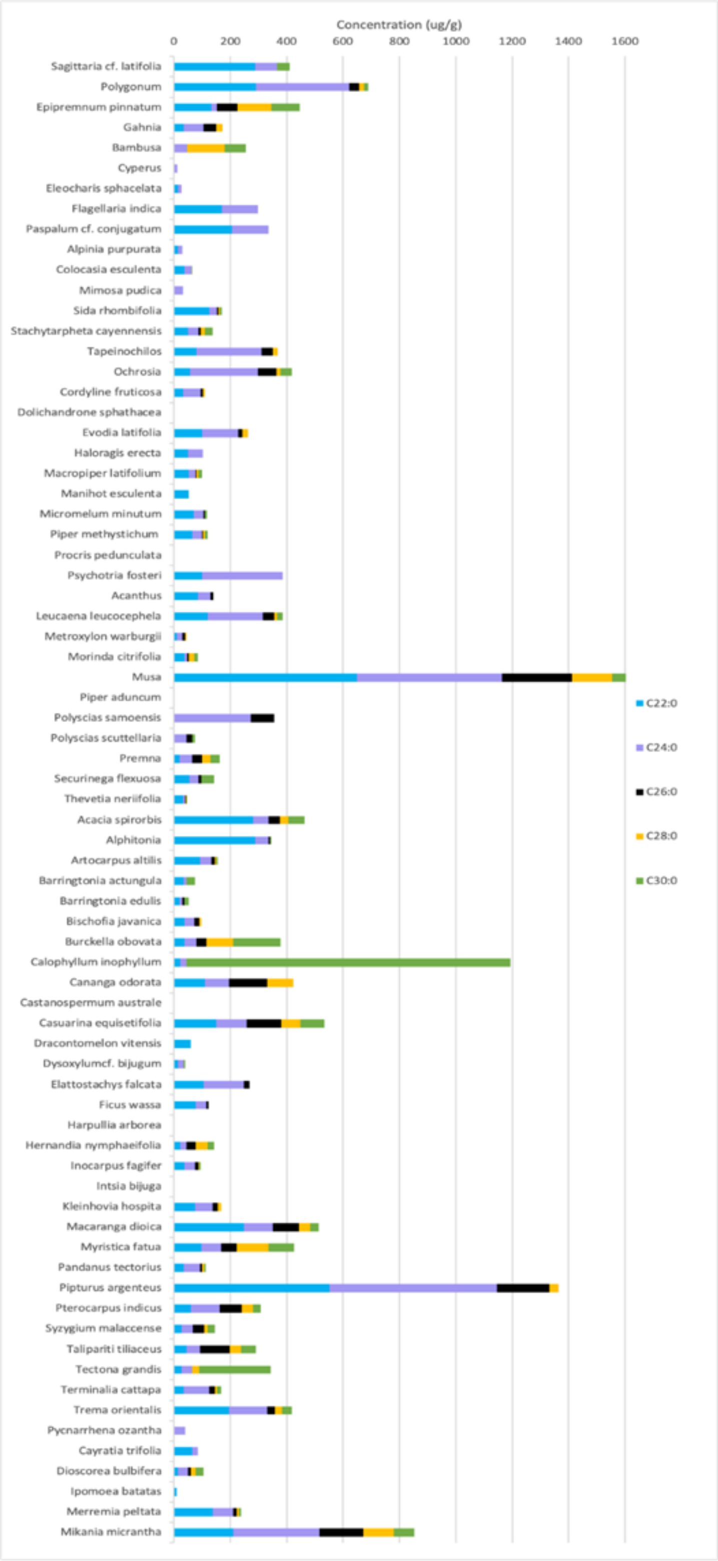

Fig. S8. Fatty acid compilation from modern plants collected from Vanuatu. Details on plant collection and lipid extraction can be found in Krentscher et al.35. The fatty acid analysis was performed as described in this study.

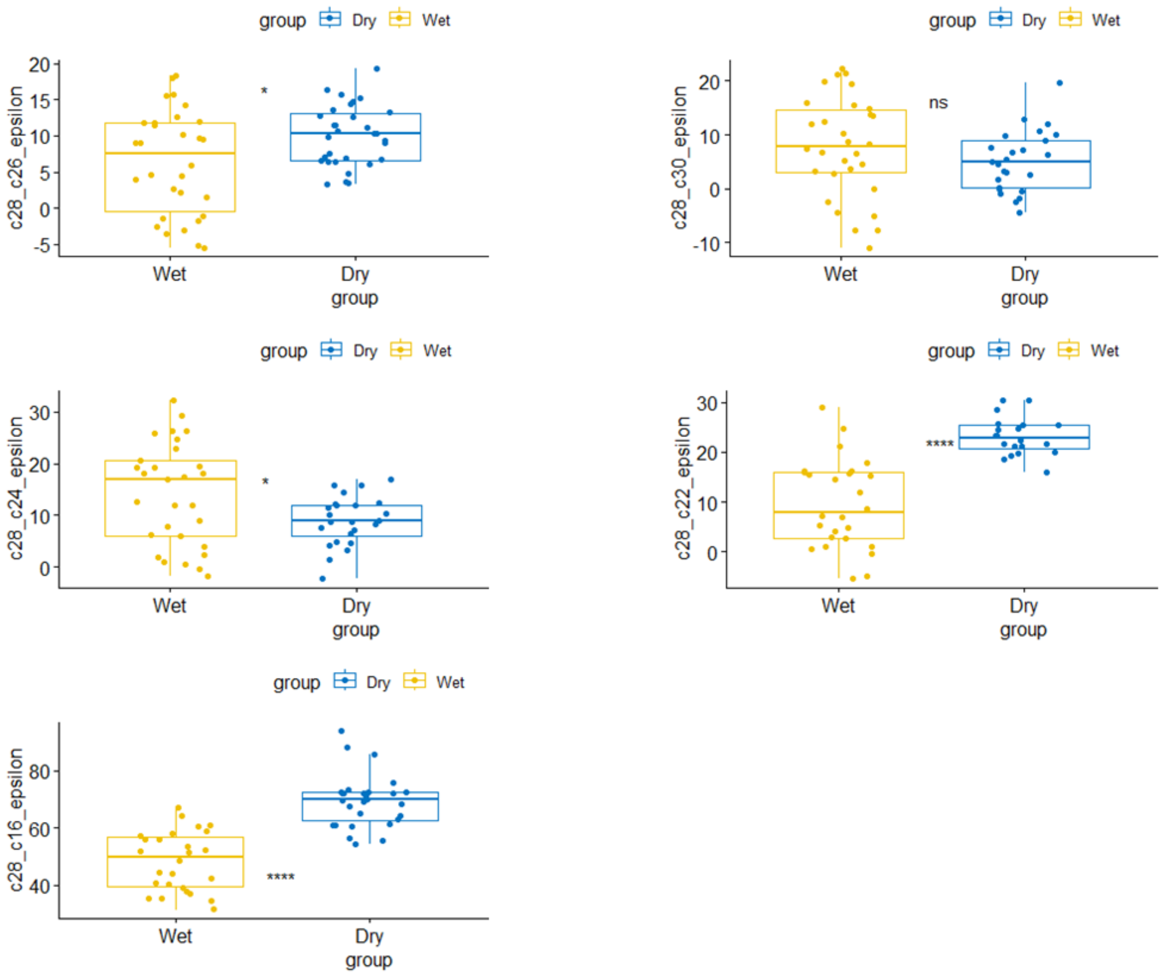

Fig. S9. Box plots for the comparison between epsilon values in the dry (blue) and wet (yellow) phase defined in figure S7. The relative offset between *n*-alkanoic acids is shown as significant (*) or non significant (ns).

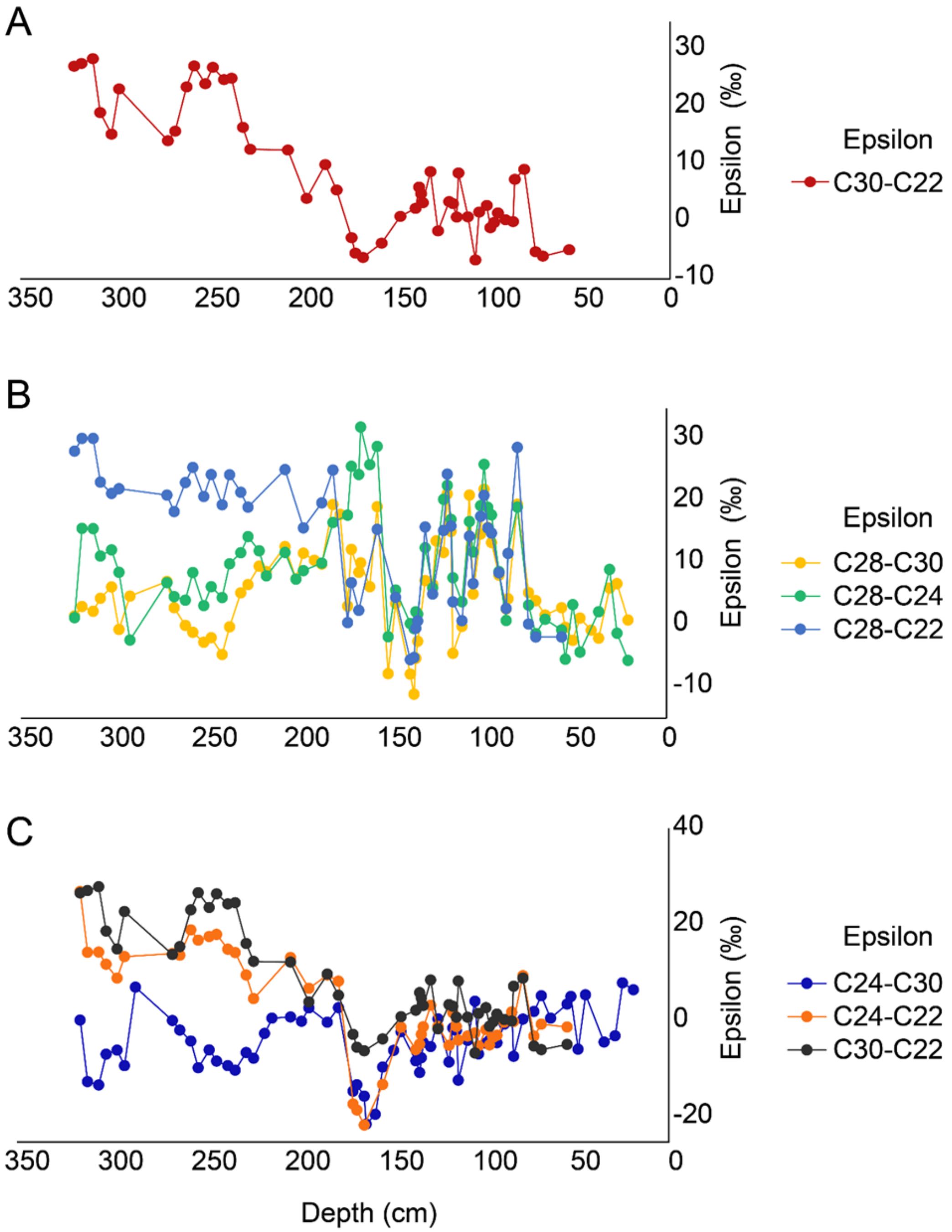

Fig. S10. Downcore relative offset between *n*-alkanoic acids expressed as epsilon values. (A) Epsilon values for *n*-C30/*n*-C22 in red. (B) Comparison of *n*-C28/*n*-C30 (yellow), *n*-C28/*n*-C24 (blue), *n*-C28/*n*-C22 (light blue) epsilon values. (C) Comparison of *n*-C24/*n*-C30 (dark blue), *n*-C24/*n*-C22 (light blue), *n*-C30/*n*-C22 (light red) epsilon values.

Table S1. List of radiocarbon dates, including the Laboratory code of the Ion Beam Physics of ETH Zurich, the material dated, the core depths, uncalibrated ^{14}C ages and error, $F^{14}C$ values and error, and the $\delta^{13}C$ ‰ of the samples. Samples with $\delta^{13}C$ < 15 ‰ (in red), roots (*), and postbomb dates were excluded from the age model.

Lab code	Material	Depth (cm)	14C age BP	14C age BP ± error	F14C	F14C ± error	δ 13C ‰
112215.1.1	plant remain	5	-15955	16	1.22	0.0149	-28.9
112216.1.1	plant remain *	17	-407	533	1.05	0.0069	-26.7
112218.1.1	plant remain	27	216	54	1.12	0.0125	-25.9
112217.1.1	plant remain	27	-882	177	0.97	0.0066	-27.8
112219.1.1	plant remain	37	245	17	0.97	0.0094	-26.7
132318.1.1	plant remain	42	2566	63	0.74	0.0057	-12.0
112220.1.1	plant remain *	47	-287	53	1.04	0.0069	-29.0
132319.1.1	plant remain	52	2156	62	0.77	0.0060	-18.7
112222.1.1	plant remain	57	1743	18	0.80	0.0064	-26.9
112221.1.1	plant remain	57	1773	18	0.80	0.0065	-22.7
132321	plant remain	58	1859	22	0.79	0.0022	-23.3
132321	plant remain	58	2152	22	0.77	0.0021	-18.5

112223.1.1	plant remain	66	2334	19	0.75	0.0056	-16.4
132322	plant remain	68	2642	23	0.72	0.0020	-14.4
101676.1.1	plant remain*	71	264	49	0.96	0.0053	-26.6
101675.1.1	plant remain*	71	378	43	0.98	0.0074	-26.2
101677.1.1	plant remain*	71	430	17	0.95	0.0021	-21.0
132323.1.1	organic matter	79	2965	63	0.74	0.0057	-8.8
132324	organic matter	83	2815	23	0.70	0.0020	-13.8
112224.1.1	plant remain*	87	-201	17	1.03	0.0105	-24.3
132325	organic matter	93	2742	23	0.71	0.0020	-11.7
132326.1.1	organic matter	96	3025	66	0.69	0.0056	-7.3
112225.1.1	plant remain*	97	-354	18	1.05	0.0109	-24.0
112226.1.1	plant remain	107	2131	60	0.77	0.0057	-31.2
112227.1.1	uncertain	116	3016	19	0.69	0.0047	-15.6
112228.1.1	plant remain	121	2520	19	0.73	0.0053	-23.3
112229.1.1	plant remain	135	2398	19	0.74	0.0055	-27.3
112230.1.1	plant remain	137	2321	18	0.75	0.0056	-27.1

101678.1.1	uncertain	141	2813	48	0.71	0.0045	-19.4
112231.1.1	plant remain	147	2480	26	0.73	0.0054	-21.1
112232.1.1	plant remain	157	2514	19	0.73	0.0053	-28.0
112234.1.1	plant remain	167	2463	19	0.74	0.0054	-27.8
112235.1.1	plant remain	173	2621	20	0.72	0.0052	-27.0
112237.1.1	plant remain	181	5769	20	0.49	0.0024	-28.5
112238.1.1	plant remain	197	2933	24	0.69	0.0048	-23.5
112239.1.1	plant remain	201	2781	59	0.71	0.0052	-29.3
101679.1.1	plant remain	211	2894	48	0.70	0.0042	-26.7
112240.1.1	plant remain	217	3023	63	0.69	0.0054	-28.7
112241.1.1	plant remain	227	3169	19	0.67	0.0045	-30.7
112242.1.1	plant remain	237	3035	19	0.69	0.0047	-29.2
112243.1.1	plant remain	251	3132	22	0.68	0.0046	-25.5
112244.1.1	plant remain	251	3142	18	0.68	0.0046	-28.8
112245.1.1	plant remain	261	3896	20	0.62	0.0038	-23.0
112246.1.1	plant remain	271	3359	19	0.66	0.0043	-29.9

101681.1.1	uncertain	281	3906	59	0.66	0.0016	-30.5
101680.1.1	plant remain	281	3286	19	0.61	0.0046	-32.1
112248.1.1	plant remain	291	3982	20	0.61	0.0037	-21.9
112249.1.1	plant remain	307	3370	19	0.66	0.0043	-30.2
112250.1.1	plant remain	317	3561	21	0.64	0.0041	-29.4
132327	plant remain	346	4834	23	0.55	0.0016	5.2
101683.1.1	uncertain	351	4916	68	0.46	0.0013	-5.5
101682.1.1	plant remain	351	6212	22	0.54	0.0068	-26.2
132328.1.1	organic material	361	4377	69	0.59	0.0050	-22.0
132329.1.1	plant remain	378	4115	66	0.61	0.0050	-28.0
132330.1.1	plant remain	391	5083	70	0.54	0.0046	-18.8
132331.1.1	plant remain	421	3937	67	0.62	0.0052	-22.1
101684.1.1	plant remain	425	3023	88	0.69	0.0147	-27.7

Table S2. List of sterols and ketone considered in this study with number of carbon atoms, origin, and selected ions (underlined the ions quantified), and standard details including producer, CAS number, and standard lot number.

**Compound**	**C atoms**	**Origin**	**Target m/z**	**Standard details**
Coprostanol	C27	Human feces	215,<br>355,<br>370	Sigma-Aldrich<br>CAS 360-68-9<br>Lot 0000188007
Epicoprostanol	C27	Epimerization of coprostanol	215,<br>355,<br>370	Sigma-Aldrich<br>CAS 516-92-7<br>Lot 127M4099V
Cholesterol	C27	Zoosterol	329,<br>368	Sigma-Aldrich<br>CAS 57-88-5<br>Lot SLBR2606V
Cholestanol	C27	Zoosterol	370,<br>455	Avanti<br>80-97-7<br>Lot 700064P-5MG-J-011
Palmitone	C31	*Colocasia esculenta* schott	71,<br>239,<br>255	abcr GmbH, 16-Hentriacontanone;<br>lot 1398514

Supplementary References

1. Rothwell, R. G. & Croudace, I. w. Twenty Years of XRF Core Scanning Marine Sediments: What Do Geochemical Proxies Tell Us? in *Micro-XRF Studies of Sediment Cores: Applications of a non-destructive tool for the environmental sciences* (eds. Croudace, I. W. & Rothwell, R. G.) 25–102 (Springer Netherlands, Dordrecht, 2015). doi:10.1007/978-94-017-9849-5_2.

2. Wirrmann, D., Eagar, S. H., Harper, M. A., Leroy, É. & Sémah, A.-M. First insights into mid-Holocene environmental change in central Vanuatu inferred from a terrestrial record from Emaotfer Swamp, Efaté Island. *Quaternary Science Reviews* **30**, 3908–3924 (2011).

3. D'Anjou, R. M., Bradley, R. S., Balascio, N. L. & Finkelstein, D. B. Climate impacts on human settlement and agricultural activities in northern Norway revealed through sediment biogeochemistry. *Proc. Natl. Acad. Sci. U.S.A.* **109**, 20332–20337 (2012).

4. Keenan, B. *et al.* Molecular evidence for human population change associated with climate events in the Maya lowlands. *Quaternary Science Reviews* **258**, 106904 (2021).

5. Raposeiro, P. M. *et al.* Climate change facilitated the early colonization of the Azores Archipelago during medieval times. *Proc. Natl. Acad. Sci. U.S.A.* **118**, e2108236118 (2021).

6. Sear, D. A. *et al.* Human settlement of East Polynesia earlier, incremental, and coincident with prolonged South Pacific drought. *Proc. Natl. Acad. Sci. U.S.A.* **117**, 8813–8819 (2020).

7. Shillito, L.-M. *et al.* Pre-Clovis occupation of the Americas identified by human fecal biomarkers in coprolites from Paisley Caves, Oregon. *Sci. Adv.* **6**, eaba6404 (2020).

8. White, A. J. *et al.* Fecal stanols show simultaneous flooding and seasonal precipitation change correlate with Cahokia's population decline. *Proc. Natl. Acad. Sci. U.S.A.* **116**, 5461–5466 (2019).

9. Prost, K., Birk, J. J., Lehndorff, E., Gerlach, R. & Amelung, W. Steroid Biomarkers Revisited – Improved Source Identification of Faecal Remains in Archaeological Soil Material. *PLoS ONE* **12**, e0164882 (2017).

10. Larson, G. *et al.* Phylogeny and ancient DNA of *Sus* provides insights into neolithic expansion in Island Southeast Asia and Oceania. *Proc. Natl. Acad. Sci. U.S.A.* **104**, 4834–4839 (2007).

11. Argiriadis, E. *et al.* Lake sediment fecal and biomass burning biomarkers provide direct evidence for prehistoric human-lit fires in New Zealand. *Sci Rep* **8**, 12113 (2018).

12. Meyers, P. A. & Ishiwatari, R. Lacustrine organic geochemistry—an overview of indicators of organic matter sources and diagenesis in lake sediments. *Organic Geochemistry* **20**, 867–900 (1993).

13. Mermoud, F., Gülaçar, F. O. & Buchs, A. 5α (H)-Cholestan-3α-ol in sediments: Characterization and geochemical significance. *Geochimica et Cosmochimica Acta* **49**, 459–462 (1985).

14. Nishimura, M. & Koyama, T. The occurrence of stanols in various living organisms and the behavior of sterols in contemporary sediments. *Geochimica et Cosmochimica Acta* **41**, 379–385 (1977).

15. Green, G., Skerratt, J. H., Leeming, R. & Nichols, P. D. Hydrocarbon and coprostanol levels in seawater, sea-ice algae and sediments near Davis station in eastern Antarctica: A regional survey and preliminary results for a field fuel spill experiment. *Marine Pollution Bulletin* **25**, 293–302 (1992).

16. Gallant, L. R. *et al.* A 4,300-year History of Dietary Changes in a Bat Roost Determined From a Tropical Guano Deposit. *Journal of Geophysical Research: Biogeosciences* **126**, e2020JG006026 (2021).

17. Venkatesan, M. I. & Santiago, C. A. Sterols in ocean sediments: novel tracers to examine habitats of cetaceans, pinnipeds, penguins and humans. *Mar. Biol.* **102**, 431–437 (1989).

18. Sachse, D. et al. Molecular Paleohydrology: Interpreting the Hydrogen-Isotopic Composition of Lipid Biomarkers from Photosynthesizing Organisms. *Annu. Rev. Earth Planet. Sci.* **40**, 221–249 (2012).

19. Ladd, S. N. et al. Leaf Wax Hydrogen Isotopes as a Hydroclimate Proxy in the Tropical Pacific. Journal of Geophysical Research: Biogeosciences 126, e2020JG005891 (2021).

20. Dansgaard, W. Stable isotopes in precipitation. Tellus 16, 436–468 (1964).

21. Risi, C., S. Bony, and F. Vimeux. Influence of convective processes on the isotopic composition (δ18O and δD) of precipitation and water vapor in the tropics: 2. Physical interpretation of the amount effect, J. Geophys. Res., 113, D19306, (2008) doi:10.1029/2008JD009943.

22. Conroy, J. L., K. M. Cobb, and D. Noone. Comparison of precipitation isotope variability across the tropical Pacific in observations and SWING2 model simulations, J. Geophys. Res. Atmos., 118, 5867–5892, (2013) doi:10.1002/jgrd.50412.

23. Garcin, Y. et al. Hydrogen isotope ratios of lacustrine sedimentary n-alkanes as proxies of tropical African hydrology: Insights from a calibration transect across Cameroon. Geochimica et Cosmochimica Acta 79, 106–126 (2012).

24. Gao, L., Edwards, E. J., Zeng, Y. & Huang, Y. Major Evolutionary Trends in Hydrogen Isotope Fractionation of Vascular Plant Leaf Waxes. PLOS ONE 9, e112610 (2014).

25. Baan, J., Holloway-Phillips, M., Nelson, D. B. & Kahmen, A. Species and biosynthetic effects cause uncorrelated variation in oxygen and hydrogen isotope compositions of plant organic compounds. Geochimica et Cosmochimica Acta (2023) doi:10.1016/j.gca.2023.04.013.

26. Aggarwal, P. K. et al. Proportions of convective and stratiform precipitation revealed in water isotope ratios. Nature Geosci 9, 624–629 (2016).

27. Kurita, N. Water isotopic variability in response to mesoscale convective system over the tropical ocean. Journal of Geophysical Research: Atmospheres 118, 10,376-10,390 (2013).

28. Risi, C. et al. Process-evaluation of tropospheric humidity simulated by general circulation models using water vapor isotopic observations: 2. Using isotopic diagnostics to understand the mid and upper tropospheric moist bias in the tropics and subtropics. Journal of Geophysical Research: Atmospheres 117, (2012).

29. Scholl, M. A., Ingebritsen, S. E., Janik, C. J. & Kauahikaua, J. P. Use of Precipitation and Groundwater Isotopes to Interpret Regional Hydrology on a Tropical Volcanic Island: Kilauea Volcano Area, Hawaii. Water Resources Research 32, 3525–3537 (1996).

30. Freeman, K. H. & Pancost, R. D. Biomarkers for Terrestrial Plants and Climate. in *Treatise on Geochemistry* 395–416 (Elsevier, 2014). doi:10.1016/B978-0-08-095975-7.01028-7.

31. Volkman, J., Johns, R., Gillan, F., Perry, G. & Bavor Jr, H. Microbial lipids of an intertidal sediment—I. Fatty acids and hydrocarbons. *Geochimica et cosmochimica acta* **44**, 1133–1143 (1980).

32. Van Bree, L. *et al.* Seasonal variability in the abundance and stable carbon-isotopic composition of lipid biomarkers in suspended particulate matter from a stratified equatorial lake (Lake Chala, Kenya/Tanzania): Implications for the sedimentary record. *Quaternary Science Reviews* **192**, 208–224 (2018).

33. Ladd, S. N., Nelson, D. B., Schubert, C. J. & Dubois, N. Lipid compound classes display diverging hydrogen isotope responses in lakes along a nutrient gradient. Geochimica et Cosmochimica Acta 237, 103–119 (2018).

34. Blaauw, Maarten, and J. Andrés Christen. Flexible paleoclimate age-depth models using an autoregressive gamma process. (2011): 457-474.

35. Krentscher, C., Dubois, N., Camperio, G., Prebble, M. & Ladd, S. N. Palmitone as a potential species-specific biomarker for the crop plant taro (Colocasia esculenta Schott) on remote Pacific islands. *Organic Geochemistry* **132**, 1–10 (2019).
