# Supplementary material for: Sedimentary biomarkers of human presence and taro cultivation reveal early horticulture in Remote Oceania
Source: Commun Earth Environ. 2024 Nov 6;5(1):667. doi: 10.1038/s43247-024-01831-8 (PMC11541199; doi:10.1038/s43247-024-01831-8)
Supplement: Supplementary file 3 — Reporting Summary [file 43247_2024_1831_MOESM3_ESM.pdf]

nature portfolio

Corresponding author(s): Giorgia Camperio

Last updated by author(s): Jun 4, 2024

Reporting Summary

Nature Portfolio wishes to improve the reproducibility of the work that we publish. This form provides structure for consistency and transparency in reporting. For further information on Nature Portfolio policies, see our Editorial Policies and the Editorial Policy Checklist.

Statistics

For all statistical analyses, confirm that the following items are present in the figure legend, table legend, main text, or Methods section.

n/a	Confirmed	
☒	☐	The exact sample size (*n*) for each experimental group/condition, given as a discrete number and unit of measurement
☒	☐	A statement on whether measurements were taken from distinct samples or whether the same sample was measured repeatedly
☒	☐	The statistical test(s) used AND whether they are one- or two-sided<br>*Only common tests should be described solely by name; describe more complex techniques in the Methods section.*
☒	☐	A description of all covariates tested
☐	☐	A description of any assumptions or corrections, such as tests of normality and adjustment for multiple comparisons
☒	☐	A full description of the statistical parameters including central tendency (e.g. means) or other basic estimates (e.g. regression coefficient) AND variation (e.g. standard deviation) or associated estimates of uncertainty (e.g. confidence intervals)
☒	☐	For null hypothesis testing, the test statistic (e.g. *F*, *t*, *r*) with confidence intervals, effect sizes, degrees of freedom and *P* value noted<br>*Give P values as exact values whenever suitable.*
☐	☒	For Bayesian analysis, information on the choice of priors and Markov chain Monte Carlo settings
☒	☐	For hierarchical and complex designs, identification of the appropriate level for tests and full reporting of outcomes
☒	☐	Estimates of effect sizes (e.g. Cohen's *d*, Pearson's *r*), indicating how they were calculated

Our web collection on statistics for biologists contains articles on many of the points above.

Software and code

Policy information about availability of computer code

Data collection	no software
Data analysis	R Statistical Software (v4.2.0, R core team 2022

For manuscripts utilizing custom algorithms or software that are central to the research but not yet described in published literature, software must be made available to editors and reviewers. We strongly encourage code deposition in a community repository (e.g. GitHub). See the Nature Portfolio guidelines for submitting code & software for further information.

Data

Policy information about availability of data

All manuscripts must include a data availability statement. This statement should provide the following information, where applicable:

- Accession codes, unique identifiers, or web links for publicly available datasets
- A description of any restrictions on data availability
- For clinical datasets or third party data, please ensure that the statement adheres to our policy

Supplementary Materials are available for this paper. All data needed to evaluate the conclusions in the paper are present in the paper and the Supplementary Materials. The dataset underlying the study are deposited in the ETH Zurich research collection at the following reserved DOI https://doi.org/10.3929/ethz-b-000652386 and will be made available upon publication.

Research involving human participants, their data, or biological material

Policy information about studies with human participants or human data. See also policy information about sex, gender (identity/presentation), and sexual orientation and race, ethnicity and racism.

Reporting on sex and gender	*Use the terms sex (biological attribute) and gender (shaped by social and cultural circumstances) carefully in order to avoid confusing both terms. Indicate if findings apply to only one sex or gender; describe whether sex and gender were considered in study design; whether sex and/or gender was determined based on self-reporting or assigned and methods used. Provide in the source data disaggregated sex and gender data, where this information has been collected, and if consent has been obtained for sharing of individual-level data; provide overall numbers in this Reporting Summary. Please state if this information has not been collected. Report sex- and gender-based analyses where performed, justify reasons for lack of sex- and gender-based analysis.*
Reporting on race, ethnicity, or other socially relevant groupings	*Please specify the socially constructed or socially relevant categorization variable(s) used in your manuscript and explain why they were used. Please note that such variables should not be used as proxies for other socially constructed/relevant variables (for example, race or ethnicity should not be used as a proxy for socioeconomic status). Provide clear definitions of the relevant terms used, how they were provided (by the participants/respondents, the researchers, or third parties), and the method(s) used to classify people into the different categories (e.g. self-report, census or administrative data, social media data, etc.) Please provide details about how you controlled for confounding variables in your analyses.*
Population characteristics	*Describe the covariate-relevant population characteristics of the human research participants (e.g. age, genotypic information, past and current diagnosis and treatment categories). If you filled out the behavioural & social sciences study design questions and have nothing to add here, write "See above."*
Recruitment	*Describe how participants were recruited. Outline any potential self-selection bias or other biases that may be present and how these are likely to impact results.*
Ethics oversight	*Identify the organization(s) that approved the study protocol.*

Note that full information on the approval of the study protocol must also be provided in the manuscript.

Field-specific reporting

Please select the one below that is the best fit for your research. If you are not sure, read the appropriate sections before making your selection.

☐ Life sciences ☐ Behavioural & social sciences ☒ Ecological, evolutionary & environmental sciences

For a reference copy of the document with all sections, see nature.com/documents/nr-reporting-summary-flat.pdf

Ecological, evolutionary & environmental sciences study design

All studies must disclose on these points even when the disclosure is negative.

Study description	A paleoecological study was conducted on a 425 cm long peat core retrieved from the Emaotfer pond on the island of Vanuatu (lat. 17°47'6.66'' S, long. 168°23'55.22'' E). Sedimentary biomarkers (fecal molecules, palmitone, leaf waxes) and sedimentological proxies (XRF, TOC%, grain size) were measured in the sediment samples.
Research sample	One-centimeter sediment samples were sliced from the 425 cm long peat core approximately every 5 cm. These samples were chosen to be distributed along the length of the core. The core was retrieved from the Emaotfer swamp near the Teouma archaeological site to allow for a comparison between the paleoecological findings and the archaeological knowledge from the site.
Sampling strategy	The deepest core section retrieved reached 425 cm in depth and was subsampled at 1 cm resolution following XRF scanning and samples were then freeze-dried. Macrofossils were separated for radiocarbon measurements. The sub-sample was then split with 1.5 cm3 used for bulk analyses while the rest of the sediment was used for biomarker extraction (up to ~3 g of dried sediment).
Data collection	The peat core was retrieved using multiple drives of a 50 cm length Russian peat corer by Matiu Prebble assisted by Giorgia Camperio, Nathalie Dubois, S. Nemiah Ladd, and Ronald Lloren
Timing and spatial scale	The sediment core was retrieved in 30 July 2017.
Data exclusions	Only radiocarbon dates not
Reproducibility	Reporducibility is limited by the availabiltiy of sediment samples especially the material left from the sediment core retrieved in 2017. However methodological reproducibility is ensured by the established sampling/extraction/measurments protocols described in the method section.
Randomization	N/A

Blinding	Blinding is not relevant to the study, sediment samples have to be chosen on the length of the core.

Did the study involve field work? ☒ Yes ☐ No

Field work, collection and transport

Field conditions	In July 2017, we cored Emaotfer swamp using multiple drives of a 50 cm length Russian peat corer. The deepest core section retrieved reached 425 cm in depth and was subsampled at 1 cm resolution following XRF scanning and samples were then freeze-dried. Macrofossils were separated for radiocarbon measurements. The sub-sample was then split with 1.5 cm3 used for bulk analyses while the rest of the sediment was used for biomarker extraction (up to ~3 g of dried sediment).
Location	Coring location on the side of the Emaotfer swamp on the island of Efate, Vanuatu. Coring location coordinates: lat. 17°47'6.66'' S, long. 168°23'55.22'' E.). Water depth at the coring site was 0.3 m.
Access & import/export	The research permit was approved by the Vanuatu National Cultural Council and the Department of Environmental Protection and Conservation (DEPC) in June 2017.
Disturbance	Boat deployment on lakes and swamp can diffuse alochtonous species, the floating device used were thorouglly cleaned to avoid any intoduction of allochtonous species from site to site.

Reporting for specific materials, systems and methods

We require information from authors about some types of materials, experimental systems and methods used in many studies. Here, indicate whether each material, system or method listed is relevant to your study. If you are not sure if a list item applies to your research, read the appropriate section before selecting a response.

Materials & experimental systems

n/a	Involved in the study
☒	☐ Antibodies
☒	☐ Eukaryotic cell lines
☐	☒ Palaeontology and archaeology
☒	☐ Animals and other organisms
☒	☐ Clinical data
☒	☐ Dual use research of concern
☒	☐ Plants

Methods

n/a	Involved in the study
☒	☐ ChIP-seq
☒	☐ Flow cytometry
☒	☐ MRI-based neuroimaging

Palaeontology and Archaeology

Specimen provenance	Macrofossils from the retrieved sediment core. The research permit was approved by the Vanuatu National Cultural Council and the Department of Environmental Protection and Conservation (DEPC).
Specimen deposition	Sedimentology laboratory, Eawag, Dubendorf
Dating methods	Radiocarbon measurements

☒ Tick this box to confirm that the raw and calibrated dates are available in the paper or in Supplementary Information.

Ethics oversight	The Vanuatu Cultural Center provided guidance in the study protocol

Note that full information on the approval of the study protocol must also be provided in the manuscript.

Plants

Seed stocks

Report on the source of all seed stocks or other plant material used. If applicable, state the seed stock centre and catalogue number. If plant specimens were collected from the field, describe the collection location, date and sampling procedures.

Novel plant genotypes

Describe the methods by which all novel plant genotypes were produced. This includes those generated by transgenic approaches, gene editing, chemical/radiation-based mutagenesis and hybridization. For transgenic lines, describe the transformation method, the number of independent lines analyzed and the generation upon which experiments were performed. For gene-edited lines, describe the editor used, the endogenous sequence targeted for editing, the targeting guide RNA sequence (if applicable) and how the editor was applied.

Authentication

Describe any authentication procedures for each seed stock used or novel genotype generated. Describe any experiments used to assess the effect of a mutation and, where applicable, how potential secondary effects (e.g. second site T-DNA insertions, mosiacism, off-target gene editing) were examined.
